# Mutations in the Beta Propeller *WDR72* Cause Autosomal-Recessive Hypomaturation Amelogenesis Imperfecta

**DOI:** 10.1016/j.ajhg.2009.09.014

**Published:** 2009-11-13

**Authors:** Walid El-Sayed, David A. Parry, Roger C. Shore, Mushtaq Ahmed, Hussain Jafri, Yasmin Rashid, Suhaila Al-Bahlani, Sharifa Al Harasi, Jennifer Kirkham, Chris F. Inglehearn, Alan J. Mighell

**Affiliations:** 1 Leeds Institute of Molecular Medicine, St James's University Hospital, University of Leeds, LS9 7TF Leeds, UK; 2 Leeds Dental Institute, University of Leeds, LS2 9LU Leeds, UK; 3 Yorkshire Regional Genetics Service, Chapel Allerton Hospital, Leeds Teaching Hospitals NHS Trust, LS7 4SA Leeds, UK; 4 Gene Tech Lab 146/1, Shadman Jail Road, Lahore, 54000, Pakistan; 5 King Edward Medical University, Lahore, 54000, Pakistan; 6 Al-Nahda Hospital, P.O.Box: 937 Muscat, P.C. 111 Sultanate of Oman; 7 Military Dental Centre, P.O.Box: 721, Seeb, P.C. 111, Sultanate of Oman

## Abstract

Healthy dental enamel is the hardest and most highly mineralized human tissue. Though acellular, nonvital, and without capacity for turnover or repair, it can nevertheless last a lifetime. Amelogenesis imperfecta (AI) is a collective term for failure of normal enamel development, covering diverse clinical phenotypes that typically show Mendelian inheritance patterns. One subset, known as hypomaturation AI, is characterised by near-normal volumes of organic enamel matrix but with weak, creamy-brown opaque enamel that fails prematurely after tooth eruption. Mutations in genes critical to enamel matrix formation have been documented, but current understanding of other key events in enamel biomineralization is limited. We investigated autosomal-recessive hypomaturation AI in a consanguineous Pakistani family. A whole-genome SNP autozygosity screen identified a locus on chromosome 15q21.3. Sequencing candidate genes revealed a point mutation in the poorly characterized *WDR72* gene. Screening of *WDR72* in a panel of nine additional hypomaturation AI families revealed the same mutation in a second, apparently unrelated, Pakistani family and two further nonsense mutations in Omani families. Immunohistochemistry confirmed intracellular localization in maturation-stage ameloblasts. WDR72 function is unknown, but as a putative β propeller is expected to be a scaffold for protein-protein interactions. The nearest homolog, WDR7, is involved in vesicle mobilization and Ca^2+^-dependent exocytosis at synapses. Vesicle trafficking is important in maturation-stage ameloblasts with respect to secretion into immature enamel and removal of cleaved enamel matrix proteins via endocytosis. This raises the intriguing possibility that WDR72 is critical to ameloblast vesicle turnover during enamel maturation.

## Main Text

Mature dental enamel is acellular, nonvital, and without capacity for turnover or significant repair. When formed normally, it is the hardest mineralized human tissue and is able to last a lifetime. Amelogenesis, the process by which enamel is produced, is dependent on induction of epithelial-derived ameloblasts through epithelial and mesenchymal interactions^1^, ^2^, ^3^. Ameloblasts pass through a number of functional stages, including presecretion, secretion of the enamel matrix, and a brief transition stage prior to enamel maturation.^4^, ^5^, ^6^ Ameloblasts migrate from the dentino-enamel junction (DEJ) to the eventual tooth surface, where apoptosis occurs before tooth eruption.^7^ Each ameloblast gives rise to an enamel rod consisting of hydroxyapatite crystals (Ca_10_[PO_4_]_6_[OH]_2_).^8^ The physical arrangement of enamel rods is key to the strength of enamel in health.

Upon initiation of the secretion phase, the enamel matrix is laid down, consisting primarily of amelogenin (AMELX; Xp22.1-p22.3 [MIM ^∗^300391]) and other functionally important proteins, including enamelin (ENAM; 4q21 [MIM ^∗^606585]) and ameloblastin (AMBN; 4q21 [MIM ^∗^601259]).^9^, ^10^, ^11^ This matrix also includes thin hydroxyapatite crystals that probably extend across the full thickness of the enamel matrix, from the DEJ to the eventual tooth surface. During maturation, these crystals expand in width and thickness to fill the space previously occupied by the matrix proteins, which are degraded and removed, leaving mature enamel with only traces of protein on eruption.^12^, ^13^ Only two enamel-matrix-modifying enzymes have been identified. Matrix metalloproteinase 20 (MMP20; 11q22.3–q23 [MIM ^∗^604629])^14^, ^15^ has a very restricted pattern of expression and is secreted by ameloblasts into the enamel matrix at the same time as AMELX, ENAM, and AMBN. Kallikrein 4 (KLK4; 19q13.3–q13.4 [MIM ^∗^603767])^16^ is a widely expressed serine protease expressed by transition- and maturation-stage ameloblasts. Both MMP20 and KLK4 are critical to the ordered cleaving of enamel matrix proteins necessary for normal enamel biomineralization. Ameloblasts remove cleaved enamel matrix proteins through receptor-mediated endocytosis.^17^

Amelogenesis imperfecta (AI) is the name given to a clinically and genetically heterogeneous group of inherited enamel biomineralization defects characterized by failure of normal amelogenesis.^18^, ^19^ It usually involves all teeth in the primary and secondary dentitions. Subsequent early clinical tooth failure can be associated with significant patient morbidity. AI prevalence data are limited, reports of 1 in 700 and 1 in 14,000 being described in Sweden and the USA, respectively.^20^, ^21^ Mendelian patterns of inheritance are often recognized. The nosology of AI is controversial. Improved insight into the genetic causes of AI will aid future classification, which continues to be based primarily on dental phenotypes in isolation.^19^ Some syndromes, such as Jalili syndrome, exhibit enamel phenotypes indistinguishable from AI occurring alone and represent an opportunity to gain insight into key gene functions in different tissues, as well as to inform improved classification of enamel defects.^22^, ^23^, ^24^

Hypoplastic forms of AI lead to markedly diminished volumes of enamel matrix deposition. These contrast with the near-normal enamel matrix volumes, and hence near-normal tooth crown morphologies, that generally characterize hypocalcified or hypomaturation forms of AI.^18^, ^19^, ^20^ Mutations in *AMELX*^25^, ^26^, ^27^ (X-linked inheritance) or *ENAM*^10^, ^25^, ^28^, ^29^ (either autosomal-dominant or autosomal-recessive inheritance) are the recognized causes of human hypoplastic AI in isolation, but they do not account for all hypoplastic AI. Animal models indicate that other genes are also critical to enamel matrix secretion, and these remain candidate genes for human hypoplastic AI.^30^

Hypocalcified AI is distinguished by enamel that is so poorly mineralized that it can be scraped away easily and fails very rapidly. *FAM83H* mutations are the only known cause of autosomal-dominant hypocalcified AI and appear relatively common.^31^, ^32^, ^33^, ^34^ FAM83H is a cellular protein of unknown function expressed during presecretory and secretory ameloblast stages.^35^ Its recent discovery has confirmed that processes not taking place in the extracellular enamel matrix are also critical to amelogenesis.

Hypomaturation AI is typified by weak, opaque, and discolored enamel that lacks normal translucency and inappropriately retains enamel matrix proteins. Clinical distinction from hypocalcified AI is difficult after posteruptive changes that occur once tooth crowns are in the mouth and begin to fail. Three mutations have been described in *MMP20*, and one in *KLK4*, as causes of autosomal-recessive hypomaturation AI.^14^, ^15^, ^16^, ^36^ A single *AMELX* mutation (p.P41T) also causes hypomaturation AI with reduced AMELX-MMP20 interactions.^37^

Null mouse models for *Klk4* and *Mmp20* have been reported.^38^, ^39^, ^40^ The enamel in *Klk4* null mice was of normal thickness and had decussating enamel rods as expected. However, retained enamel matrix proteins delayed but did not prevent significant levels of enamel maturation. The gross enamel failure when eating even a soft diet was primarily attributed to failure of enamel crystallites to grow together, interlock, and function as a unit. By contrast, *Mmp20* null mice have enamel that is thinner and less-mineralized than normal and lacks the expected enamel rod organization.^41^ It, too, fails prematurely through chipping away and attrition. Although the critical nature of *MMP20*, *KLK4*, and *AMELX* mutations to hypomaturation AI are known, it is also recognized that other causes of hypomaturation AI remain to be discovered.^42^, ^43^

We identified and recruited ten families with autosomal-recessive hypomaturation AI and origins from either Kashmir in Pakistan (P1-P5) or Oman (O1-O5). The study was performed in accordance with the principles of the declaration of Helsinki, with informed patient consent, and with ethical approval in the UK, Pakistan, and Oman. When possible, peripheral blood samples were obtained from affected and unaffected family members and genomic DNA was prepared by a conventional salting method. Alternatively, DNA samples were prepared from Oragene (DNA Genotek) saliva collections in accordance with the manufacturers instructions. Dental examinations were undertaken.

**Figure 1:**
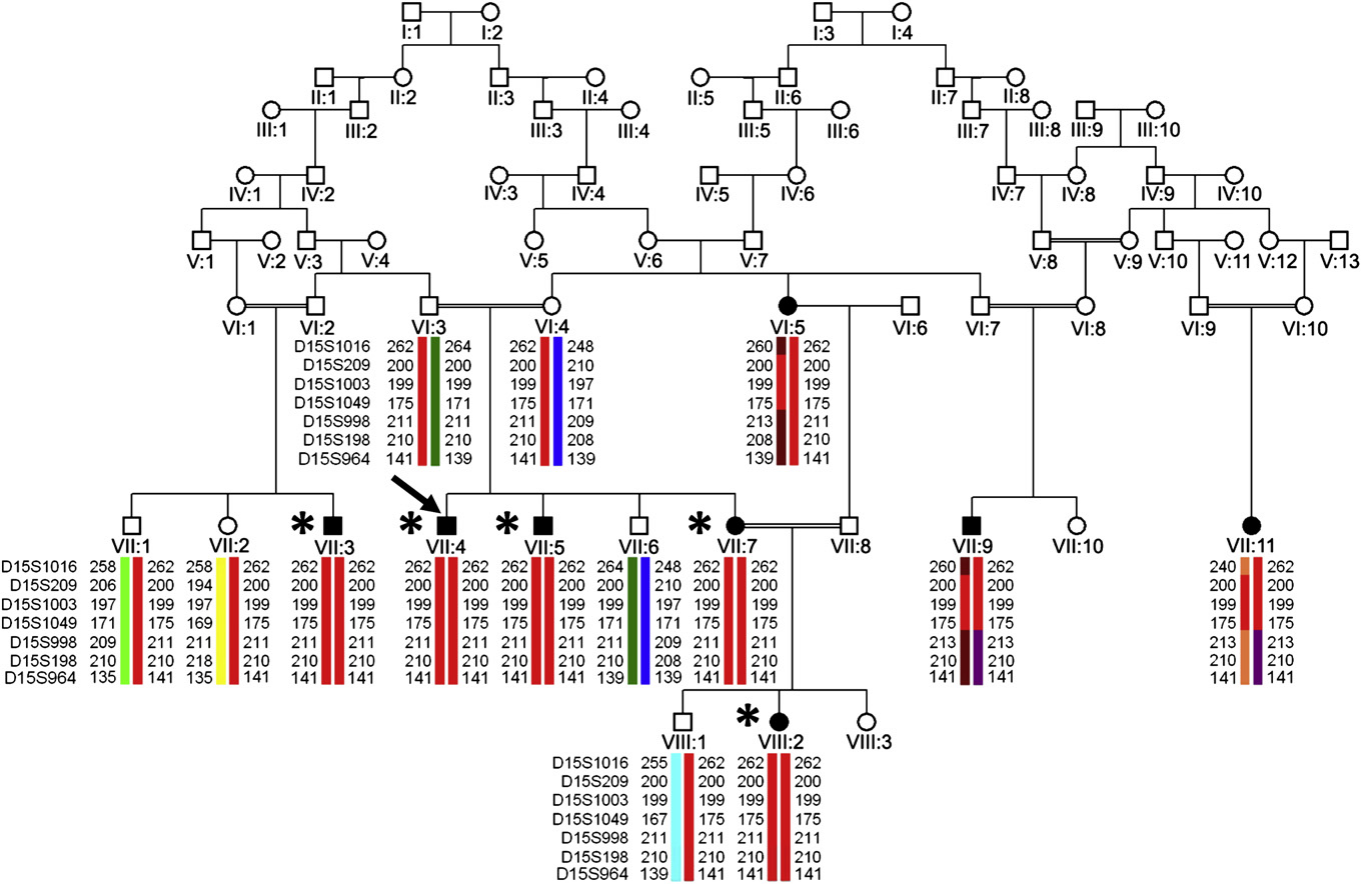
Pedigree and Haplotypes of Family P1 Segregating Autosomal-Recessive Hypomature AI Solid circles (female) and squares (male) distinguish affected individuals, the proband being marked with an arrow. Microsatellite marker genotypes used for defining the candidate interval are recorded, the red bars defining the marker haplotype that tracks with the affected status. Each individual included in the whole-genome screen is marked by an asterisk (^∗^).

**Figure 2:**
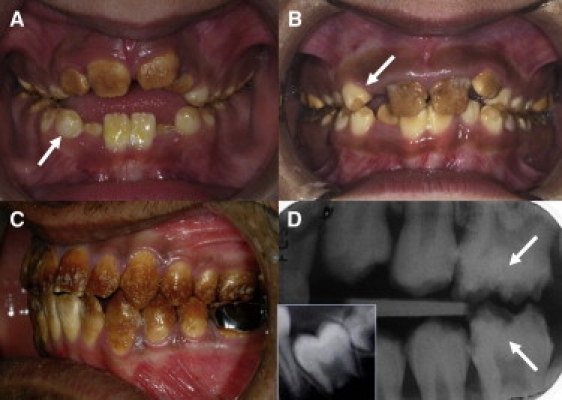
Clinical Appearances of Hypomaturation AI in Patients with *WDR72* Mutations (A and B) The dentitions of an 8-year-old (A; family P1) and a 7-year-old (B; family P2), illustrating opaque, creamy-brown, discolored enamel that starts to chip away (arrows) from the underlying dentine soon after eruption and leaves an irregular, stained surface. The upper permanent incisor teeth are more markedly affected than are the lower teeth. An anterior open bite, which does not segregate with the AI, is observed in (A) but not (B). (C) The dentition of a 19-year-old member of family P1 illustrates posteruption changes, including loss of surface enamel and marked brown staining, with relative sparing of the lower incisor teeth and cervical enamel. (D) A bitewing radiograph of the individual illustrated in (C), demonstrating gross posteruptive loss of enamel that leaves an irregular surface that is particularly evident on molar teeth (arrow). There is an obvious lack of radiodensity between enamel and dentine in comparison to the inset image of an unerupted tooth from an individual without AI, which has a typical curved crown morphology and enamel (arrow head) that is distinct from the dentine (^∗^). Note: the inset image is reproduced at approximately two-thirds the size of the affected teeth.

The AI in the largest of these families, P1 (Figure 1), was generalized with involvement of the primary and secondary dentitions (Figure 2). The clinical appearances of the teeth on eruption and radiographic appearances prior to eruption were consistent with near-normal enamel matrix volume formation. However, at the time of eruption, the enamel was creamier and more opaque than that of normal teeth. Once in the mouth, the enamel soon began to undergo posteruptive changes, including variable degrees of brown discoloration and loss of enamel tissue. In some instances enamel chipped away, but attrition was also evident. Variable malocclusions were observed, and one affected individual had an anterior open bite. This malocclusion has been reported sporadically in AI of different genetic causes, and the underlying mechanisms remain unknown.^44^ Teeth were sensitive to thermal and physical stimuli. The clinical features were consistent with hypomaturation AI. No other health problems segregated with AI.

**Figure 3:**
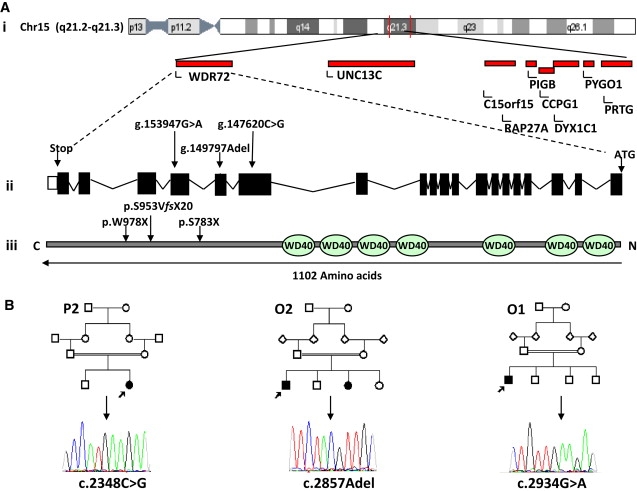
*WDR72* Mutations and P2, O1, and O2 Family Pedigrees (A) (i) Schematic representation of the annotated genes at the proximal end of the AI-linked interval on chromosome 15q21.3. (ii) Exonic structure (boxes) of *WDR72* with the start (ATG) and stop codons marked and with introns represented by lines. The positions of the mutations are marked with arrows. (iii) Representation of the protein structure with approximate positions of the WD40 domains marked. Positions of the mutations in the wild-type protein are marked with arrows. (B) Pedigrees for families P2, O1, and O2 with sequences of the mutations found in each. A diamond shape represents an individual of unknown sex.

DNA samples from five affected individuals from family P1 were mixed in equimolar amounts and then subjected to Affymetrix 50K SNP microarray analysis (Figure 1). A large region of homozygozity on chromosome 15 was identified between SNPs rs727614 at 51,032,409 bp and rs2350482 at 87,909,765 bp (Table S1, available online). Twenty polymorphic microsatellite markers within the region were then genotyped in all family members available at that time, these being the sibships to the left of the pedigree and excluding individuals VI:5, VII:9, and VII:11 (Table S2). The resultant genotypes were analyzed with GeneMapper V4.0 software (Applied Biosystems) (Figure 1). Two-point linkage analyses performed with the MLINK program revealed LOD scores of up to 3.3 (Table S3). Multipoint linkage analysis with markers D15S1016, D15S998, and D15S964 against AI gave a maximum LOD score of 4.1 at D15S1016. Subsequently, DNA samples were obtained from the three additional family members—VI:5, VII:9, and VII:11—and these were genotyped with the same set of markers, giving further refinement. The locus spanned approximately 3 cM or 4.4 Mb between D15S1016 and D15S998 and contained 16 annotated genes (Figure 3, Table S4).

The coding exons and intron-exon junctions of all 16 genes were amplified by PCR and sequenced by Big Dye Terminator v3.1 Cycle Sequencing Kit on an ABI 3130XL DNA Analyzer. The only deleterious mutation identified was in *WDR72* (Figure 3, Tables S4 and S5). A point mutation identified in exon 15 (c.2348C>G) is predicted to introduce a premature stop codon (p.S783X).

All coding exons of *WDR72*, as well as surrounding intronic sequences, were then amplified by PCR and sequenced in affected individuals from the remaining hypomaturation AI families (P2–P5 and O1–O5). Family P2, which is not known to be related to P1, shared the same p.S783X mutation and had a similar clinical phenotype (Figure 2). An additional point mutation was identified in exon 17 (c.2934G>A) in family O1 (Figure 3). This results in another premature stop codon, p.W978X. In family O2, a deletion of a single base pair in exon 16 (c.2857Adel.) leads to a terminal frame shift (p.S953V*fs*X20). The clinical appearances of the affected individuals in families O1 and O2 were consistent with those observed in the other two families. Each mutation cosegregated consistently with the disease phenotype. The mutation in families P1 and P2 was absent from 192 normal Pakistani individuals, and the mutations in families O1 and O2 were not identified in 192 normal individuals of differing ethnic origins. No mutations were identified in the remaining six families. This is consistent with the previous observation that hypomaturation AI is likely to be genetically heterogeneous.^42^, ^43^

**Figure 4:**
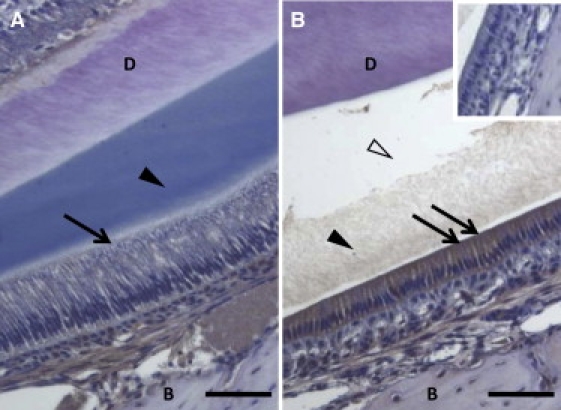
WDR72 Localization during Mouse Incisor Development in Secretory and Maturation Phases WDR72 immunoreactivity is observed in the enamel organ, with more intense staining in maturation ameloblasts (double arrow in panel B) than in secretory ameloblasts (arrow in panel A). Black arrowheads and the open arrowhead mark the enamel matrix and the enamel space after demineralization, respectively. D and B denote dentine and bone, respectively. Inset in (B) shows negative control in which primary antibody has been omitted. Scale bar represents 100 μm.

For investigation of the localization of WDR72 during amelogenesis, polyclonal antisera were raised commercially in rabbits to the synthetic peptide CETGTLERHETGERA (WDR72 amino acids 587–600 with a terminal C added) (GeneScript). Immunohistochemistry was undertaken in accordance with standard methods on sections of formalin-fixed and paraffin-embedded demineralized mouse jaws that included erupting incisor teeth. Dewaxed and rehydrated sections were incubated with the primary polyclonal antisera for 1 hr. Immunoreactivity was visualized with horseradish peroxidase and DAB (Dako EnVision). WDR72 immunoreactivity was observed in maturation-stage ameloblasts (Figure 4), as well as in some bone and connective-tissue cells. This is consistent with database transcript records that indicate widespread expression in different tissue types.

The *WDR72* gene consists of 19 coding exons spanning around 250 kb, encoding a protein of 1102 amino acids about which remarkably little is known (Figure 3). Predictive algorithm analyses identify seven WD40 domains within the first 600 amino acids from the N terminus. WD40 domains are characterized by minimally conserved runs of about 40 amino acids typically bracketed by glycine-histidine and tryptophan-aspartic acid (GH-WD). This domain was first identified in the β subunit of the heterotrimeric GTP-binding protein (G protein), which also has seven WD40 domains.^45^ However, a large and functionally diverse range of eukaryotic proteins with different numbers of WD40 domains have subsequently been described.^46^ WD40 domains are believed to form the blades of β propeller structures with four to eight blades, but most typically with seven blades.^47^ Propeller blade surfaces act as multisite docking platforms critical for transient protein-complex formation.^48^ Regulatory functions for WD40 domain proteins include histone and/or chromatin modification,^49^ ubiquitin-mediated proteolysis,^50^ cell signaling,^51^ and vesicle turnover,^52^ among many others.

The functions of WDR72 are unknown. Its closest human homolog and a possible paralog is WDR7, also known as Rabconnectin-3β or TGF-β resistance-associated protein (TRAG).^53^, ^54^ It exhibits 37% homology (58% similarity) to WDR72 and also contains seven WD40 domains. WDR7 (Rabconnectin-3β) and DMXL2 (Rabconnectin-3α), another WD40-domain protein, are the two subunits of Rabconnectin.^55^, ^56^ Rabconnectin contributes to activation and deactivation of RAB3A, a small GTP-binding protein that is a positive regulator of vesicle mobilization and Ca^2+^ dependent exocytosis of neurotransmitter release at synaptic vesicles.^55^, ^57^

Vesicle turnover is important in maturation-stage ameloblasts. They continue to have secretory functions, including the secretion of KLK4 and Amelotin (AMTN; 4q13.3 [MIM ^∗^610912]) into the enamel matrix.^40^, ^58^ The ameloblasts undergo regular cyclical morphological changes at the cell-enamel matrix interface. Cleaved enamel matrix proteins are removed by endocytosis that involves multiple cell-membrane invaginations and a “ruffle-ended” ameloblast morphology, although the mechanisms remain poorly understood.^3^, ^17^ In part, the morphological changes relate to essential cyclical pH changes.^59^, ^60^ The possibility that WDR72 has a similar role in Ca^2+^-dependent vesicle turnover is therefore intriguing, but there are currently no data supporting this idea that WDR72 and WDR7 function in similar ways.

The unexpected identification of *WDR72* mutations as a cause of autosomal-recessive hypomaturation AI creates an opportunity for additional insight into the understanding of amelogenesis and biomineralization. Furthermore, given the localization of WDR72 in different tissue types, this discovery may also stimulate research in other fields.

## Web Resources

The URLs for data presented herein are as follows:

Online Mendelian Inheritance in Man (OMIM), http://ncbi.nlm.nih.gov/Omim/
National Center for Biotechnology Information (NCBI) human gene master map for chromosome 15, ==http://www.ncbi.nlm.nih.gov/projects/mapview/maps.cgi?taxid=9606&chr=15

## Supplemental Data

Document S1. Five Tables
