# Supplementary material for: Mutations in the Beta Propeller WDR72 Cause Autosomal-Recessive Hypomaturation Amelogenesis Imperfecta
Source: Am J Hum Genet. 2009 Nov 13;85(5):699–705. doi: 10.1016/j.ajhg.2009.09.014 (PMC2775821; doi:10.1016/j.ajhg.2009.09.014)
Supplement: Document S1. Five Tables [file mmc1.pdf]

Supplemental Data

AJHG, Volume *85*

Mutations in the β-Propeller *WDR72* Cause Autosomal-Recessive Hypomaturation Amelogenesis Imperfecta

Walid El-Sayed, David A. Parry, Roger C. Shore, Mushtaq Ahmed, Hussain Jafri, Yasmin Rashid, Suhaila Al-Bahlani, Sharifa Al Harasi, Jennifer Kirkham, Chris F. Inglehearn, and Alan J. Mighell

Table S1. Genotyping by Affymetrix Genome-Wide Human 50K SNP Array

SNP genotypes in the vicinity of the linked region on chromosome 15. The refined region of homozygosity between rs727614 and rs2350482 is highlighted in blue. SNPs with one or more "No call" have been deleted for clarity.

15	AB	rs10519143	45842859
15	AB	rs684715	45865024
15	BB	rs4775714	45865217
15	AA	rs10519145	45885244
15	BB	rs10519146	45885715
15	AA	rs1453862	45928633
15	BB	rs751467	46034246
15	AB	rs2934192	46045683
15	AA	rs2469583	46146164
15	AA	rs964611	46384806
15	AB	rs10519170	46473467
15	AA	rs2015637	46504145
15	AB	rs2466792	46540437
15	AB	rs10519177	46544487
15	AB	rs7176364	46567381
15	AB	rs612617	46693869
15	AA	rs9302143	46843406
15	AA	rs10519189	46850721
15	AA	rs8029015	46920821
15	AA	rs10519191	46941541
15	AA	rs10519192	46950212
15	BB	rs10519194	46950338
15	BB	rs10519195	46950476
15	AA	rs10519196	46951793
15	AB	rs2009404	47004015
15	AA	rs10519206	47137711
15	AA	rs2059935	47381025
15	AA	rs10519234	47720436
15	AA	rs10519238	47756591

15	BB	rs1484555	47801369
15	AA	rs690671	47835966
15	AA	rs10519239	47873772
15	AA	rs2933551	47876236
15	AB	rs10519242	47935182
15	BB	rs10519243	47974477
15	AA	rs10519248	48003494
15	BB	rs4359385	48006512
15	BB	rs10519251	48056965
15	BB	rs2413992	48057872
15	AA	rs10519252	48070953
15	AB	rs2414007	48124729
15	BB	rs6493426	48375850
15	AA	rs16963644	48425969
15	AB	rs720599	48451807
15	AB	rs10519276	48543532
15	AB	rs8037553	48577525
15	BB	rs493728	48678247
15	AB	rs10519284	48790334
15	AA	rs10519285	48810031
15	AA	rs2414052	48927593
15	BB	rs10519295	49319939
15	AA	rs10519296	49320386
15	AA	rs17647478	49325648
15	AB	rs2008691	49335602
15	AB	rs7175922	49434029
15	AB	rs2446405	49434085
15	AA	rs10519314	49764451
15	BB	rs10519315	49764503
15	AA	rs10519316	49764604
15	BB	rs10519317	49764885
15	BB	rs10519318	49764955
15	AB	rs10519319	49766074
15	AA	rs10519320	49766117
15	AA	rs10519321	49767401
15	AA	rs3765067	49778943
15	AA	rs10519324	49853117
15	AA	rs2554324	49935371
15	AA	rs2554309	49967342
15	BB	rs17540338	50145825
15	BB	rs2414128	50157020
15	AA	rs10518677	50217065
15	AA	rs8030407	50314794
15	BB	rs4776028	50329088
15	AA	rs7164872	50411155
15	BB	rs16964915	50440565
15	AA	rs10518683	50441415
15	AA	rs10518684	50445664
15	BB	rs1405184	50468669
15	AA	rs10518687	50474725
15	BB	rs10518689	50489692
15	AA	rs1615235	50514364
15	BB	rs4776055	50630148
15	AA	rs3751613	50689206

15	AB	rs2899492	50720554
15	AA	rs1991300	50756038
15	AA	rs2414172	50756247
15	BB	rs10518694	50859965
15	BB	rs7178476	50950376
15	AA	rs7161750	50951074
15	AB	rs1899734	50964781
15	AB	rs1873811	51000577
15	AA	rs716188	51032169
15	AB	rs727614	51032409
15	AA	rs4776110	51047697
15	BB	rs2263563	51072643
15	BB	rs10518701	51108037
15	BB	rs2899511	51109121
15	AA	rs1392859	51159739
15	BB	rs1508032	51160525
15	AA	rs557171	51171992
15	BB	rs608531	51172430
15	BB	rs562235	51173102
15	BB	rs10518704	51213979
15	AA	rs1508028	51240406
15	AA	rs10518706	51240801
15	AA	rs10518711	51257387
15	AA	rs473488	51257760
15	BB	rs579584	51258236
15	BB	rs1508022	51293730
15	BB	rs558995	51295622
15	BB	rs566369	51295884
15	BB	rs479747	51296193
15	AA	rs482541	51296486
15	BB	rs2414230	51385015
15	AA	rs1352172	51385736
15	AA	rs8037429	51404201
15	AA	rs750486	51404276
15	AB	rs2651529	51422551
15	AA	rs2134002	51422868
15	BB	rs10518720	51454847
15	AA	rs10518721	51489390
15	BB	rs613246	51517424
15	AA	rs10518724	51595524
15	AA	rs4451891	51675833
15	BB	rs7171915	51677914
15	AA	rs7171619	51677951
15	BB	rs7178691	51686598
15	AA	rs1906402	51693562
15	AA	rs1906404	51693694
15	BB	rs1906405	51694486
15	AA	rs2926882	51721345
15	AA	rs10518731	51723710
15	AA	rs689811	51725856
15	BB	rs10518732	51726334
15	BB	rs10518733	51727599
15	AA	rs1621807	51735154
15	AA	rs989790	51759463

15	AA	rs578431	51899114
15	AA	rs580129	51899315
15	AA	rs1478199	51948262
15	BB	rs1478200	51948279
15	BB	rs1020944	51960753
15	BB	rs2711592	52058975
15	BB	rs2616911	52063145
15	BB	rs991264	52063843
15	AA	rs2616910	52063875
15	BB	rs10518747	52067223
15	BB	rs2616907	52067663
15	BB	rs7168151	52135477
15	AA	rs1897025	52151283
15	BB	rs10518755	52151396
15	BB	rs2414273	52151635
15	AB	rs2414270	52175915
15	AA	rs9302174	52324587
15	BB	rs10518762	52325052
15	BB	rs573320	52465606
15	AA	rs573740	52470723
15	AA	rs579664	52574650
15	BB	rs1850996	52656746
15	BB	rs8028630	52662763
15	AA	rs746321	52662853
15	AA	rs2681962	52685879
15	AA	rs1851006	52691684
15	AA	rs8042622	52692250
15	BB	rs2460605	52693011
15	BB	rs10518774	52700466
15	BB	rs9302185	52742156
15	BB	rs992348	52887050
15	AA	rs10518782	52908228
15	BB	rs10518783	52908472
15	BB	rs16975367	52908612
15	BB	rs10518784	52908811
15	BB	rs1875405	52908961
15	BB	rs10518785	52909004
15	BB	rs1875404	52909198
15	AA	rs1875403	52909283
15	AA	rs10518786	52909418
15	BB	rs1392978	52910643
15	AA	rs1915204	52923662
15	BB	rs2202085	52924242
15	BB	rs2414385	52927040
15	AA	rs10518788	52927196
15	AA	rs10518789	52929651
15	AA	rs10518790	52930043
15	BB	rs10518791	52930076
15	BB	rs1915215	52986769
15	AA	rs1629366	53040846
15	BB	rs10518792	53042160
15	BB	rs1728866	53134392
15	AA	rs1115333	53134745
15	BB	rs2414391	53135583

15	AA	rs10518797	53169953
15	AA	rs491004	53184152
15	BB	rs7178925	53184680
15	AA	rs3809540	53276704
15	BB	rs3809539	53276906
15	AA	rs10518805	53307474
15	BB	rs8041885	53452381
15	BB	rs8041044	53452605
15	AA	rs10518810	53452798
15	AA	rs1075938	53577983
15	AA	rs10518817	53740892
15	BB	rs2414428	53794987
15	BB	rs2303580	53940164
15	BB	rs10518824	53947068
15	BB	rs7174459	53995275
15	BB	rs10518830	53998510
15	BB	rs10518831	54004605
15	AA	rs2036740	54077312
15	BB	rs2036741	54077390
15	BB	rs2414455	54077691
15	AA	rs7494783	54108894
15	AA	rs2584689	54271580
15	AA	rs10518839	54271655
15	AA	rs795793	54523500
15	BB	rs10518850	54523570
15	BB	rs6493858	54523770
15	BB	rs10518855	54616277
15	BB	rs10518870	54805180
15	AA	rs10518871	54805516
15	AA	rs10518872	54809475
15	BB	rs8035474	54810002
15	BB	rs1010699	54837904
15	AA	rs1477475	54839561
15	AA	rs715338	55003159
15	AA	rs213154	55012890
15	BB	rs213168	55028949
15	AA	rs2962994	55129991
15	AA	rs10518898	55277897
15	BB	rs10518899	55278474
15	BB	rs935329	55297253
15	AA	rs10518904	55317767
15	BB	rs17820119	55343090
15	BB	rs2431032	55353151
15	AA	rs8034843	55368601
15	AA	rs10518909	55391871
15	AA	rs10518910	55392566
15	BB	rs2243451	55407479
15	BB	rs4534776	55408068
15	BB	rs10518911	55420610
15	AA	rs1664456	55469145
15	BB	rs1664450	55472780
15	BB	rs4774941	55520686
15	AA	rs10518922	55569541
15	AA	rs1280367	55572415

15	AA	rs10518923	55573538
15	AA	rs1280368	55573712
15	AA	rs7181170	55655740
15	AA	rs1868243	55667810
15	BB	rs937250	55685484
15	BB	rs10518940	55927236
15	AA	rs10518943	55942641
15	AA	rs10518944	55942766
15	AA	rs7167150	55945899
15	AA	rs10518947	55998943
15	BB	rs1550574	56000660
15	BB	rs2414525	56014925
15	BB	rs4646607	56072680
15	AA	rs4646590	56090429
15	AA	rs4646588	56091605
15	AA	rs4646587	56092075
15	AA	rs4646584	56092694
15	BB	rs4646583	56093843
15	BB	rs1400216	56185894
15	BB	rs2414535	56220582
15	AA	rs4775014	56231848
15	BB	rs1618930	56255258
15	BB	rs1711055	56256876
15	AA	rs10518968	56380218
15	AA	rs10518969	56381289
15	AA	rs1973310	56381475
15	AA	rs402345	56432498
15	BB	rs8036597	56436960
15	BB	rs10518972	56437647
15	AA	rs10518976	56538493
15	AA	rs10518977	56543441
15	AA	rs10518978	56543465
15	AA	rs10518982	56587046
15	AA	rs10518984	56615162
15	AA	rs9302203	56721161
15	BB	rs7180487	56726482
15	AA	rs6494031	56726560
15	BB	rs1054991	56936692
15	AA	rs10518990	56974110
15	AA	rs9302206	57096765
15	BB	rs10518996	57164738
15	AA	rs1044673	57176758
15	AA	rs10518998	57188191
15	BB	rs10519003	57498029
15	AA	rs10519004	57498583
15	AA	rs10519005	57498983
15	BB	rs10519039	57780050
15	BB	rs4775203	57835458
15	AA	rs8037497	57896190
15	AA	rs10519032	57940963
15	AA	rs1437480	57942057
15	AA	rs10519031	57970297
15	AA	rs10519030	57970388
15	AA	rs10519029	58024231

15	BB	rs10519028	58046059
15	AA	rs10519027	58046413
15	BB	rs10519026	58046468
15	AA	rs10519025	58046764
15	BB	rs6494167	58222639
15	AA	rs1481845	58222843
15	AA	rs7176165	58246247
15	AA	rs338418	58304602
15	AA	rs1876868	58344233
15	AA	rs1033028	58404227
15	BB	rs10519043	58456322
15	BB	rs1869489	58561404
15	AA	rs2033721	58609267
15	BB	rs920201	58610323
15	BB	rs10519051	58610595
15	BB	rs10519052	58610709
15	AA	rs10519053	58618217
15	BB	rs4774367	58619313
15	AA	rs930351	58619369
15	AA	rs2414677	58655419
15	BB	rs1054156	58718763
15	BB	rs1647978	58726541
15	BB	rs339998	58735055
15	BB	rs1657800	58753207
15	BB	rs10519058	58754219
15	AA	rs3909594	58755653
15	AA	rs9302215	58847659
15	AA	rs7164773	58855240
15	BB	rs10519067	58855639
15	BB	rs10519068	58855996
15	AA	rs10519070	58856045
15	AA	rs8041822	58892849
15	AA	rs6494227	58893410
15	AA	rs10519080	58925751
15	AA	rs1680481	58934236
15	BB	rs10519084	58934621
15	AA	rs10519085	58940843
15	AA	rs1482059	58942148
15	AA	rs1902618	58951491
15	BB	rs7174236	58970994
15	AA	rs877863	58982098
15	AA	rs2414689	59051547
15	AB	rs1523526	59051579
15	BB	rs7176717	59071701
15	AA	rs9302221	59081387
15	AA	rs10519099	59082118
15	BB	rs10519100	59082184
15	AA	rs10519105	59105685
15	BB	rs10519106	59105723
15	AA	rs8033470	59106432
15	AA	rs7171287	59159271
15	AA	rs4775359	59175796
15	AA	rs10519113	59229742
15	AA	rs1370430	59259898

15	AA	rs1529991	59272291
15	AA	rs10519115	59272497
15	BB	rs7177846	59278332
15	AA	rs3884558	59490071
15	AA	rs3935962	59611593
15	BB	rs10519122	59671428
15	BB	rs10519120	59679793
15	AA	rs1124856	59721111
15	AA	rs4350523	59721266
15	BB	rs2249195	59745321
15	BB	rs2249197	59745355
15	BB	rs10519127	59745459
15	BB	rs2414739	59781426
15	AA	rs10519131	59788424
15	AA	rs1047471	59810655
15	BB	rs10519135	59855772
15	BB	rs10519144	60015600
15	AA	rs1030861	60151984
15	AA	rs1030859	60152224
15	AA	rs10519154	60161818
15	BB	rs4775458	60162328
15	BB	rs10519155	60162745
15	BB	rs10519156	60163090
15	AA	rs10519157	60168922
15	BB	rs1898329	60170381
15	AA	rs4114259	60170457
15	AA	rs2414768	60359341
15	AA	rs290297	60483363
15	BB	rs289139	60484419
15	BB	rs10519148	60507655
15	AA	rs885188	60581199
15	AA	rs2456462	60581548
15	BB	rs10519172	60663521
15	AA	rs1463806	60839037
15	BB	rs10519179	60911880
15	AA	rs10519180	60913859
15	AA	rs10519190	61336117
15	BB	rs1017545	61341047
15	BB	rs10519204	61387929
15	AA	rs289806	61500402
15	AA	rs10519209	61500553
15	BB	rs10519210	61524978
15	AA	rs982078	61610594
15	AA	rs10519223	61722202
15	AA	rs10519213	61779744
15	BB	rs925251	61962177
15	AA	rs1471282	61999328
15	AA	rs1470315	62001337
15	AA	rs3843699	62424144
15	AA	rs3848148	62424589
15	BB	rs534969	62690114
15	BB	rs10519272	62714104
15	BB	rs10519273	62714224
15	AA	rs2019185	63287488

15	BB	rs10519294	63638524
15	AB	rs10519300	63775690
15	AA	rs934542	63803230
15	AA	rs10519303	63817519
15	BB	rs10519304	63818078
15	AA	rs10518678	64220003
15	AA	rs997204	64220250
15	BB	rs899079	64267270
15	BB	rs10518685	64401976
15	BB	rs10518686	64402527
15	BB	rs7164579	64629063
15	AA	rs1530196	64629324
15	AA	rs1866750	64694597
15	BB	rs755451	64822005
15	AA	rs9302239	64927196
15	BB	rs7183187	65021394
15	AA	rs10518705	65150337
15	BB	rs10518707	65152676
15	BB	rs10518723	65510668
15	BB	rs2589985	65632144
15	BB	rs10518735	65678272
15	BB	rs2899726	65694008
15	AA	rs4776946	65694344
15	AA	rs1878702	65744428
15	BB	rs10518738	65801304
15	BB	rs10518739	65802025
15	AB	rs10518744	65850029
15	BB	rs4539549	65857673
15	AA	rs12593698	65866615
15	BB	rs1489595	66164180
15	AA	rs10518758	66254147
15	AA	rs964691	66413596
15	BB	rs898586	66415655
15	BB	rs3816612	66438234
15	AA	rs1380881	66438517
15	AA	rs2018282	66567317
15	BB	rs10518768	66567635
15	AA	rs10518769	66567837
15	BB	rs1373697	66577067
15	BB	rs16952446	66784528
15	BB	rs10518781	66784567
15	AA	rs2924633	66881693
15	BB	rs8033662	66909768
15	BB	rs10518802	67164289
15	AA	rs311898	67164600
15	BB	rs4116076	67278521
15	BB	rs10518813	67399889
15	BB	rs10518814	67442600
15	BB	rs937724	67519790
15	AA	rs10518818	67520080
15	AA	rs1857744	67566377
15	BB	rs1820414	67656901
15	AA	rs10518821	67669717
15	AA	rs1115635	67719223

15	AA	rs4776472	67793927
15	BB	rs4632084	67836478
15	BB	rs305050	67876852
15	BB	rs747131	67877228
15	BB	rs87757	67877685
15	BB	rs10518840	67928430
15	AA	rs305002	67928959
15	AA	rs304962	67955329
15	BB	rs304949	67962776
15	AA	rs10518880	68279029
15	AA	rs7179693	68282545
15	BB	rs10518882	68287654
15	AA	rs10518912	68649182
15	BB	rs8034258	68722216
15	AA	rs10518921	68793963
15	BB	rs10518927	68893022
15	AA	rs2119538	69023413
15	BB	rs1561694	69035076
15	BB	rs894128	69070843
15	AA	rs10518941	69350055
15	AA	rs10518942	69351194
15	BB	rs2068040	69367415
15	BB	rs2083610	69401607
15	AA	rs4777372	69430841
15	AA	rs4777383	69471026
15	BB	rs10518959	69477995
15	AA	rs7165378	69478558
15	AB	rs1501428	69695631
15	BB	rs7169459	69746050
15	BB	rs8033100	69764500
15	BB	rs8027192	69793594
15	BB	rs1995332	69794407
15	AA	rs2291279	69907141
15	AA	rs9302260	69961665
15	BB	rs2128112	69973340
15	AA	rs10518975	69981510
15	BB	rs2306488	70079169
15	BB	rs2957749	70120662
15	AA	rs10518987	70352841
15	BB	rs10518988	70573082
15	AA	rs3842946	70616103
15	AA	rs7176235	70786157
15	BB	rs7182854	70866557
15	AA	rs1023924	71225675
15	AA	rs1564347	71225901
15	AA	rs1320241	71343855
15	BB	rs10518997	71343997
15	BB	rs7164883	71439227
15	BB	rs2899786	71452378
15	AA	rs2415152	71617713
15	AA	rs2127015	71772276
15	AA	rs7176701	71776874
15	AA	rs10519015	71913008
15	AA	rs10519016	71913817

15	BB	rs1349642	71930673
15	AA	rs1440101	72011684
15	BB	rs8041685	72012246
15	BB	rs893814	72121091
15	AA	rs10519048	72252601
15	AA	rs2198843	72788283
15	AA	rs8182037	73921656
15	BB	rs335699	73996350
15	BB	rs2133409	74063484
15	AA	rs3898352	74308825
15	AA	rs1801591	74365817
15	BB	rs10519134	74366145
15	AA	rs8029637	74374542
15	AA	rs10519141	74526126
15	BB	rs284903	74531868
15	BB	rs157776	74543758
15	BB	rs283796	74561160
15	BB	rs283795	74561298
15	AA	rs182253	74587810
15	AA	rs1446312	75199244
15	BB	rs10519158	75199512
15	AA	rs10519162	75259926
15	AA	rs10519164	75412758
15	BB	rs1565757	75444737
15	BB	rs907399	75558094
15	AA	rs964990	75643722
15	AA	rs3825847	76250541
15	AA	rs7163689	76352533
15	AA	rs10519198	76529809
15	AA	rs10519203	76601101
15	AA	rs8053	76628275
15	BB	rs951266	76665596
15	BB	rs10519205	76665846
15	AA	rs10519215	76970802
15	BB	rs997285	77134426
15	AA	rs939663	77211623
15	BB	rs10519231	77273165
15	BB	rs2010521	77274773
15	AA	rs1001460	77291934
15	BB	rs2865228	77404564
15	AA	rs1114640	77424162
15	AA	rs9302288	77462356
15	AA	rs10519241	77542342
15	BB	rs2297773	77542659
15	AA	rs3750004	77548286
15	AA	rs10519240	77560114
15	BB	rs1337557	77625440
15	AA	rs10519274	77678805
15	BB	rs10519271	77704802
15	BB	rs10519270	77705211
15	AA	rs10519269	77705433
15	BB	rs10519268	77711483
15	AA	rs10519267	77711769
15	BB	rs10519266	77714470

15	AA	rs10519265	77715278
15	AA	rs282802	77859875
15	AA	rs10519247	77995219
15	AA	rs10519277	78160743
15	AA	rs10519281	78193051
15	AA	rs3784761	78204268
15	AA	rs10519286	78370985
15	BB	rs10519291	78414963
15	BB	rs10519290	78786397
15	BB	rs1078107	78797170
15	BB	rs7162157	78871932
15	AA	rs925111	78872538
15	BB	rs1484226	78872583
15	AA	rs10519298	78971366
15	BB	rs10519307	79089321
15	AA	rs1320323	79128502
15	BB	rs4778908	79632208
15	AA	rs7176423	79691434
15	BB	rs8035453	79691464
15	BB	rs949898	79826783
15	AA	rs8024021	80043132
15	AA	rs1045508	80236961
15	BB	rs1846911	80255705
15	BB	rs10520568	80256811
15	BB	rs17158372	81144053
15	BB	rs10520577	81818542
15	AA	rs10520579	81959475
15	AA	rs10520580	81971211
15	AA	rs10520581	81992067
15	AA	rs10520575	82354035
15	AA	rs8035919	82355139
15	AA	rs10520571	82407336
15	BB	rs2010790	82570742
15	AA	rs7176688	82575861
15	AA	rs1429444	83180282
15	AA	rs289386	83382393
15	BB	rs2174218	83425660
15	AA	rs10520586	83425735
15	BB	rs10520585	83462510
15	BB	rs10520583	83639906
15	AA	rs10520600	83709406
15	BB	rs4842881	83785033
15	AA	rs4842882	83785187
15	AA	rs4360875	83841398
15	AA	rs10520591	83890008
15	BB	rs731760	83902979
15	BB	rs10520592	83915146
15	AA	rs1026720	83920460
15	BB	rs10520594	83929645
15	AA	rs2344440	83962569
15	AA	rs10520597	83971259
15	AA	rs1861856	83999565
15	AA	rs10520599	84024101
15	BB	rs1961601	84030610

15	AA	rs49364	84030935
15	BB	rs49362	84031006
15	AA	rs49363	84031033
15	AA	rs338553	84034855
15	BB	rs10520601	84159205
15	AA	rs10520606	84191767
15	BB	rs2344859	84191867
15	BB	rs4134376	84229261
15	AA	rs7167642	84407894
15	AA	rs2120650	84419700
15	BB	rs4506862	84477875
15	BB	rs10520609	84481778
15	AA	rs10520610	84502120
15	BB	rs10520611	84509342
15	BB	rs10520615	84570270
15	BB	rs7179949	84570820
15	AA	rs10520617	84608546
15	BB	rs10520618	84608765
15	AA	rs10520619	84609350
15	AA	rs8041327	84610811
15	AA	rs1988583	84622660
15	AA	rs10520620	84644249
15	AA	rs977316	84657715
15	AA	rs10520621	84657820
15	BB	rs10520622	84691378
15	BB	rs2060060	84701996
15	AA	rs10520623	84702054
15	AA	rs1122907	84708371
15	BB	rs10520625	84736924
15	AA	rs1431236	84751834
15	AA	rs8029257	84752366
15	AA	rs1835625	84779366
15	AA	rs10520627	84779592
15	BB	rs1431231	84779759
15	BB	rs10520628	84780388
15	BB	rs16977356	84802737
15	BB	rs7175159	84828318
15	BB	rs1110287	84863177
15	BB	rs2257019	84932031
15	AA	rs10520633	84932266
15	AA	rs10520635	84993863
15	AA	rs4887244	85147853
15	BB	rs10520640	85153480
15	AA	rs1452457	85185181
15	AA	rs1452456	85185317
15	BB	rs10520642	85201101
15	BB	rs9302343	85238873
15	AA	rs6496371	85262941
15	BB	rs10520647	85418531
15	BB	rs1034482	85428116
15	AA	rs1433461	85429462
15	BB	rs10520649	85477204
15	AA	rs1499735	85481241
15	AA	rs10520650	85484534

15	BB	rs10520651	85485214
15	AA	rs3866550	85509827
15	BB	rs10520612	85700134
15	AA	rs9284311	85729504
15	BB	rs1014276	85747931
15	AA	rs10520613	85754073
15	BB	rs10520655	85887415
15	BB		85892619
15	BB	rs10520656	85910263
15	BB	rs10520657	85915598
15	BB	rs10520658	85915676
15	AA	rs10520666	85958968
15	BB	rs10520668	86135284
15	BB	rs10520669	86160186
15	AA	rs1347424	86262610
15	AA	rs922231	86319499
15	BB	rs10520670	86343913
15	AA	rs10520672	86367621
15	AA	rs3784421	86434782
15	BB	rs6496469	86536314
15	AA	rs9302345	86590382
15	AA	rs10520678	86738287
15	AA	rs1000587	86851632
15	AA	rs3817428	87216251
15	AA	rs1810224	87338956
15	AB	rs293340	87402313
15	AA	rs293379	87434944
15	BB	rs293378	87435300
15	AA	rs293376	87436272
15	BB	rs10520682	87495167
15	BB	rs3743377	87621012
15	AB	rs2350482	87909765
15	BB	rs1256840	88329016
15	BB	rs8041707	88330488
15	AB	rs716175	88721170
15	AB	rs10520687	88768753
15	AB	rs8025842	88796475
15	AB	rs719473	88799068
15	AB	rs10520692	88955475
15	AB	rs7601	89310596
15	BB	rs10520700	89322158
15	AA	rs6496742	89324040
15	AA	rs10520697	89496905
15	BB	rs2239990	89624441
15	AA	rs2387108	89650358
15	AB	rs1079537	89675287
15	AA	rs10520695	89686018
15	AA	rs7178880	90512987
15	AA	rs10520703	90518434
15	AB	rs10520704	90521604
15	BB	rs10520705	90522481
15	AA	rs10520709	90635589
15	BB	rs10520710	90688998
15	AA	rs1455780	90786010

15	AB	rs1826853	90912591
15	AB	rs1826852	90912611
15	BB	rs426191	91001456
15	AA	rs1390790	91034256
15	AA	rs10520716	91292021

Table S2. P1 Family Microsatellite Genotypes

Microsatellite makers genotyped in the Pakistani family (P1). Analysis indicated linkage to 15q.21.3. The shared affected haplotype is highlighted in green showing an area of homozygosity spanning 3cM between D15S1016 and D15S998 markers. The affected individuals are highlighted in red.

Markers ID	Physical positions	VI:3	VI:4	VII:1	VII:2	VII:3	VII:4	VII:5	VII:6	VII:7	VIII:1	VIII:2	VI:5	VII:9	VII:11
D15S1032	50484442	162 158	166 156	146 166	162 162	162 166	162 166	162 166	158 156	162 166	156 162	166 166	? ?	? ?	? ?
D15S1016	50778351	262 264	262 248	257 262	257 262	262 262	262 262	262 262	264 248	262 262	255 262	262 262	260 262	240 262	260 262
D15S209	51017930	200 200	200 210	200 206	194 200	200 200	200 200	200 200	200 210	200 200	200 200	200 200	200 200	200 200	200 200
D15S1003	51698684	199 199	199 197	197 199	197 199	199 199	199 199	199 199	199 197	199 199	199 199	199 199	199 199	199 199	199 199
D15S1049	52999084	175 171	175 171	171 175	169 175	175 175	175 175	175 175	171 171	175 175	167 175	175 175	175 175	175 175	175 175
D15S998	55141886	211 211	211 209	209 211	211 211	211 211	211 211	211 211	211 209	211 211	211 211	211 211	211 213	213 213	213 213
D15S198	55750109	210 210	210 208	210 210	210 218	210 210	210 210	210 210	210 208	210 210	210 210	210 210	208 210	210 210	210 210
D15S1033	56039810	163 161	163 165	163 163	163 151	163 163	163 163	163 163	161 165	163 163	163 165	163 163	161 163	163 163	163 163
D15S148	56830245	129 129	129 135	129 135	129 135	129 129	129 129	129 129	129 135	129 129	129 129	129 129	130 136	129 129	130 130
D15S964	56873764	141 139	141 139	135 141	141 139	141 141	141 141	141 141	139 139	141 141	139 141	141 141	141 139	141 141	141 141
D15S643	57501059	210 208	210 220	210 224	210 216	210 210	210 210	210 210	208 220	210 210	210 220	210 210	210 220	210 210	210 210
D15S155	58200337	262 265	262 265	262 272	262 263	262 262	262 262	262 262	265 265	262 262	262 265	262 262	262 265	262 262	262 262
D15S1036	60274169	127 131	115 133	119 127	127 135	127 127	127 115	127 115	131 133	127 115	127 115	115 115	? ?	? ?	? ?

Table S3. Two-Point LOD Scores between Hypomaturation AI and Six Informative Microsatellite Markers, Showing LOD Score 3.53 at the Marker D15S1016 when θ = 0

Marker	0.0	0.1	0.2	0.3	0.4	0.5
D15S209	1.64	1.24	0.80	0.40	0.13	0.00
D15S998	1.12	0.86	0.58	0.31	0.10	0.00
D15S198	0.95	0.65	0.37	0.16	0.03	0.00
D15S1033	2.47	1.99	1.41	0.82	0.30	0.00
D15S1016	3.53	2.75	1.94	1.15	0.45	0.00
D15S964	3.42	2.63	1.83	1.06	0.40	0.00

Table S4. Candidate Genes Screened for Mutations

5q21.3 genes screened for mutations (arranged alphabetically). The pathogenic changes in the affected individuals for *WDR72* are shown. No novel SNPs were discovered.

Gene	Entrez Gene ID	OMIM	Pathogenic SNPs
CCPG1	9236	*611326	
CGNL1	84952	*607856	
RSL24D1	51187	-	
DYX1C1	161582	#127700	
MNS1	55329	*610766	
NEDD4	4734	*602278	
PIGB	9488	*604122	
PRTG	283659	-	
PYGO1	26108	*606902	
RAB27A	5873	*603868	
RFX7	64864	-	
TCF12	6938	*600480	
TEX9	374618	-	
UNC13C	440279	-	
WDR72	256764	-	c.2348C>G;p.S783X(P1&P2)<br>c.2857Adel;p.S976VfsX20(O1)<br>c.2934G>A; p.W978X(O2)
ZNF280D	54816	-	

Table S5. Oligonucleotide Primer Pairs Used to Amplify and Sequence *WDR72* Coding Exons and exon-Intron Junctions.

Exon	Forward	Reverse
2	5′-AGCACTGGGAGAACACATCA-3′	5′-CCTTCCACTGTCATTGCAGA-3′
3	5′-TGTGGTGTTTTTATTCTTCAAACAG-3′	5′-AAAAAGGGAAGGAAATGGAGA-3′
4	5′-AAAGAAAAACTTGGTGGCACA-3′	5′-TTTGCAAATGCCCTAAACAA-3′
5	5′-GAGAAATCTTCAATGTAGCCAAA-3′	5′-TTTCTGAATTTCTGCCCAAT-3′
6	5′-TGAAATGATTTTTACCTACCCAATTAT-3′	5′-TTTGAACTTTTGACAATAAACATGTAA-3′
7	5′-GGAAGCAGGTCTTCTCATGG-3′	5′-GACACATCATTCCATGTTCTGG-3′
8	5′-ATATTCATCTTATTGGCAGACTAGATT-3′	5′-AGCATGCAGGGATTTTTCC-3′
9	5′-AAAGTGGGAGGGGGAAAAA-3′	5′-TTTTTCAAGCCTGATTCTGTGA-3′
10	5′-GAAAATGTCAAAAGAGTGGGAAA-3′	5′-CTTGTTTGTGGTCCCATGCT-3′
11	5′-GGGAGGGTGCCTTGATATTT-3′	5′-TCTGTTGAAATTGCAGCTTTAGA-3′
12	5′-AATCTTGGGACGATTTTCTGA-3′	5′-TTGGAAATATGGGTCATTTTTACTT-3′
13	5′-TGTAAATGGGGCTTTAACAAAA-3′	5′-TCTGAAACTTTCCATCTGGTCA-3′
14	5′-TCTGTCTTATTCTGTTGGGGTAAA-3′	5′-TTGGTAGATGCAGCAGTCTCTT-3′
15a	5′-CCTTTAGCTGCTCTGCAATG-3′	5′-GGCTAGTGCCTCTGCTGAAA-3′
15b	5′-GAGCACAGTGGAGAAGAAGACA-3′	5′-CCTGGCAACATCAGTGAGAA-3′
15c	5′-GGGTCCTATTTCTTTGGGAAT-3′	5′-AGGAAGGAATGTTAAAGAGCTAAA-3′
16	5′-TGGTCATGTACCCATATCTTTGAT-3′	5′-AGAAAGACTAGTTATGTCCTTTCACAG-3′
17	5′-ACAAAGCTTCCCAGGTGATT-3′	5′-GGGGTATCCATGAACCACAG-3′
18	5′-TTTTGCAACATACCCAAGCA-3′	5′-CGTTCAGTTGCACCACCATA-3′
19	5′-GGAAATGTTTTAATGGTGTTTGC-3′	5′-ACCCCAAAGCTGTGTCAAAT-3′
20	5′-CTCCCCTCCTCTTGGTCTTT-3′	5′-AAACAAATGGCATCTTTTGGA-3′
